# Cell Imaging Counting as a Novel Ex Vivo Approach for Investigating Drug-Induced Hepatotoxicity in Zebrafish Larvae

**DOI:** 10.3390/ijms18020356

**Published:** 2017-02-08

**Authors:** Xuan-Bac Nguyen, Stanislav Kislyuk, Duc-Hung Pham, Angela Kecskés, Jan Maes, Deirdre Cabooter, Pieter Annaert, Peter De Witte, Annelii Ny

**Affiliations:** 1 Laboratory for Molecular Biodiscovery, Department of Pharmaceutical and Pharmacological Sciences, University of Leuven, O & N II Herestraat 49-box 824, 3000 Leuven, Belgium; bac.nguyenxuan@kuleuven.be (X.-B.N.); duchung.pham@kuleuven.be (D.-H.P.); angela.kecskes@aok.pte.hu (A.K.); jan.maes@kuleuven.be (J.M.); annelii.ny@kuleuven.be (A.N.); 2 Division Pharmaceutical Analysis, Department of Pharmaceutical and Pharmacological Sciences, University of Leuven, O & N II Herestraat 49-box 923, 3000 Leuven, Belgium; stanislav.kislyuk@kuleuven.be (S.K.); deirdre.cabooter@kuleuven.be (D.C.); 3 Drug Delivery and Disposition, Department of Pharmaceutical and Pharmacological Sciences, University of Leuven, O & N II Herestraat 49-box 921, 3000 Leuven, Belgium; pieter.annaert@kuleuven.be

**Keywords:** hepatotoxicity, drug-induced liver injury (DILI), zebrafish larvae, cell imaging counting, pharmacokinetics

## Abstract

Drug-induced liver injury (DILI) is the most common reason for failures during the drug development process and for safety-related withdrawal of drugs from the pharmaceutical market. Therefore, having tools and techniques that can detect hepatotoxic properties in drug candidates at an early discovery stage is highly desirable. In this study, cell imaging counting was used to measure in a fast, straightforward, and unbiased way the effect of paracetamol and tetracycline, (compounds known to cause hepatotoxicity in humans) on the amount of DsRed-labeled hepatocytes recovered by protease digestion from Tg(*fabp10a:DsRed*) transgenic zebrafish. The outcome was in general comparable with the results obtained using two reference methods, i.e., visual analysis of liver morphology by fluorescence microscopy and size analysis of fluorescent 2D liver images. In addition, our study shows that administering compounds into the yolk is relevant in the framework of hepatotoxicity testing. Taken together, cell imaging counting provides a novel and rapid tool for screening hepatotoxicants in early stages of drug development. This method is also suitable for testing of other organ-related toxicities subject to the organs and tissues expressing fluorescent proteins in transgenic zebrafish lines.

## 1. Introduction

Drug-induced liver injury (DILI) is the most common cause of liver injury affecting approximately 14–19 cases per 100,000 patient-years in Western patients. Among the patients diagnosed with DILI in a French study, 6% died and 12% required hospitalization [1]. DILI is also the most common reason for failures during the drug development process [2] and for safety-related withdrawal of drugs from the pharmaceutical market [3]. Therefore, having tools and techniques that can detect hepatotoxic properties in drug candidates at an early stage of discovery is highly desirable. Traditionally, this has been accomplished by performing in vivo studies in mice and rats by measuring the serum alanine aminotransferase levels and by histological analysis of liver sections. However, these models are time demanding, costly, and in addition interfere with the welfare of the animals. Hence there is an urgent need to identify fast and cost-efficient methods using alternative models with high human predictability that in the preclinical phase identify and exclude drugs with hepatotoxic properties.

Zebrafish (*Danio rerio*) are a promising small vertebrate animal model for use in biomedical research and represent a powerful alternative model to implement the 3R principle of humane animal research (replacement, reduction, and refinement) [4,5,6]. In the context of compound hepatotoxicity testing the model offers several advantages. First and foremost, zebrafish show a high similarity to mammals in hepatocyte metabolism and liver functionality [7] and display similar responses to hepatotoxicants [4]. In addition, the zebrafish liver is fully functional at 72 hpf [5,8], so hepatic responses after exposure of zebrafish larvae to hepatotoxicants can be monitored from an early stage onwards. Secondly, zebrafish are highly amenable to medium/high throughput screening. One adult pair of fish can produce approximately 200–300 eggs/week, resulting in access to large numbers of eggs/embryos. Moreover, the developing zebrafish larvae have a size of 1–4 mm, thus experiments can easily be performed in microtiter plates using only microgram amounts of compound.

Current hepatotoxicity testing using zebrafish includes endpoints such as liver degeneration and yolk retention [6], expression of liver-specific fatty acid binding protein (*lfabp10a*) in hepatocytes, and liver morphology using a Tg(*fabp10a:DsRed*) zebrafish transgenic line that specifically expresses fluorescent DsRed in hepatocytes [9]. Unfortunately, all these methods require blinding of the investigators to minimize bias and maximize the validity of the results. Besides, the 2D image-based results do not necessarily reflect the actual 3D changes in the liver. 

As hepatocytes account for 70%–75% of hepatic cells and carry out most of the liver functions [10], it is anticipated that the reduction in number of hepatocytes after drug exposure is a key indicator of hepatotoxicity. Presently, fluorescence-activated cell sorting (FACS) is the only method used to estimate ex vivo the number of hepatocytes present in zebrafish [11]. However, as the procedure requires single sample filtering and processing, the method is inherently slow and hence not suitable for medium/high throughput purposes.

In this study cell imaging counting (CIC) was used to quantify the amount of DsRed-labeled hepatocytes recovered from Tg(*fabp10a:DsRed*) transgenic zebrafish in a fast and straightforward way. Two compounds known to cause hepatotoxicity in humans, i.e., paracetamol and tetracycline [6], as well as two non-hepatotoxicants (sucrose and rutin) were selected for the study. The outcome was compared with results found with commonly used hepatotoxicity-testing methods that are based on assessing liver morphology.

The studies published to date only used immersion as the route of exposing zebrafish to drugs investigating their hepatotoxic effects. This situation can result in false-negatives due to poor absorption as observed when investigating cardiotoxicity [12]. We therefore also explored the applicability of using yolk and pericardial injection as alternative administration routes for testing hepatoxicants. Finally, we also studied the pharmacokinetics of paracetamol and tetracycline in zebrafish larvae using the concerned administration routes.

## 2. Results 

### 2.1. Determination of Maximum Non-Toxic Drug Concentration/Dose (MC/MD)

**Figure 1:**
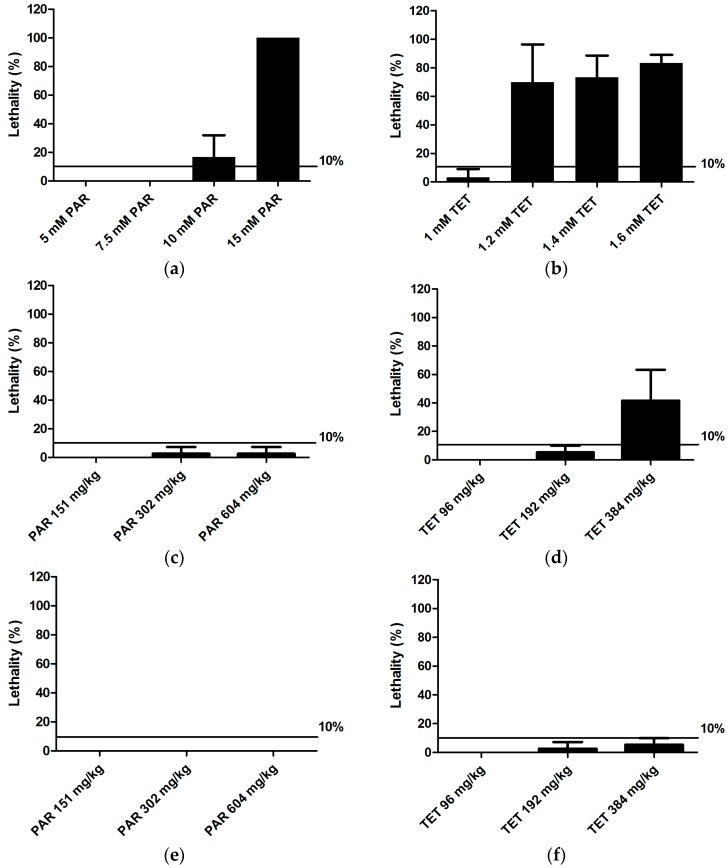
Lethality in Tg(*fabp10a:DsRed*) zebrafish larvae after immersion (**a**,**b**); yolk injection (**c**,**d**) and pericardial injection (**e**,**f**) of paracetamol (**a**,**c**,**e**) and tetracycline (**b**,**d**,**f**). Analysis was performed after three days of treatment (immersion) or three days after treatment (injection), at 6 days post-fertilization (dpf). A total of 10 and 12 larvae were used individually per condition for immersion and injections, respectively. The experiment was performed in triplicate and data were pooled. The results are expressed as percentage of dead larvae relative to the total larvae (mean ± SD). PAR: paracetamol; TET: tetracycline.

Using a range of concentrations/doses, we first determined the maximum non-toxic drug concentration (MC, in case of immersion) or dose (MD, in case of injection), i.e., the highest concentration/dose of compound causing insignificant larval death (less than 10%; see Figure 1) after three days of treatment (immersion), or three days after treatment (injection), at 6 days post-fertilization (dpf). In the case that an insignificant number of larval deaths were induced, the highest MC that resulted in a clear solution (no precipitation) or MD that could be injected without precipitation was used.

Our results show that the two hepatotoxicants (paracetamol, tetracycline) caused larval death in several of the conditions tested (Figure 1), whereas the non-hepatotoxicants (sucrose and rutin) did not induce lethality even at the highest soluble concentration/dose (results not shown). Based on these results, the MC/MD of each compound was determined. The MC or MD thus obtained, as well as two serial dilutions (50%, 25%) were used as concentrations/doses (Table 1, Table 2 and Table 3) for further hepatotoxicity testing.

### 2.2. Hepatotoxicity Assessed by Visual Analysis of Liver Morphology Using Fluorescence Microscopy

**Figure 2:**
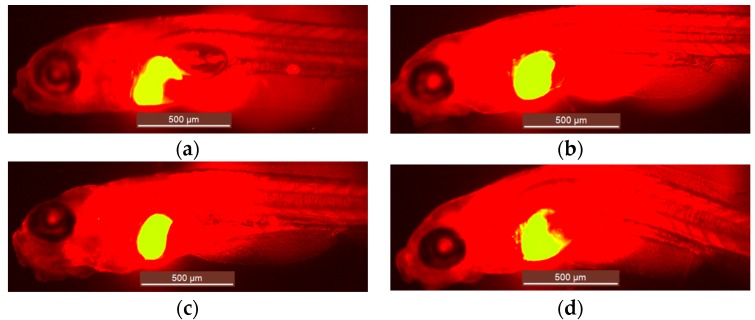
Visual analysis of liver morphology using Tg(*fabp10a:DsRed*) zebrafish larvae by fluorescence microscopy (original 40× magnification). Larvae with normal liver morphology (**a**); and abnormal liver morphology (**b**–**d**). The liver area is identified as the yellow area.

**Figure 3:**
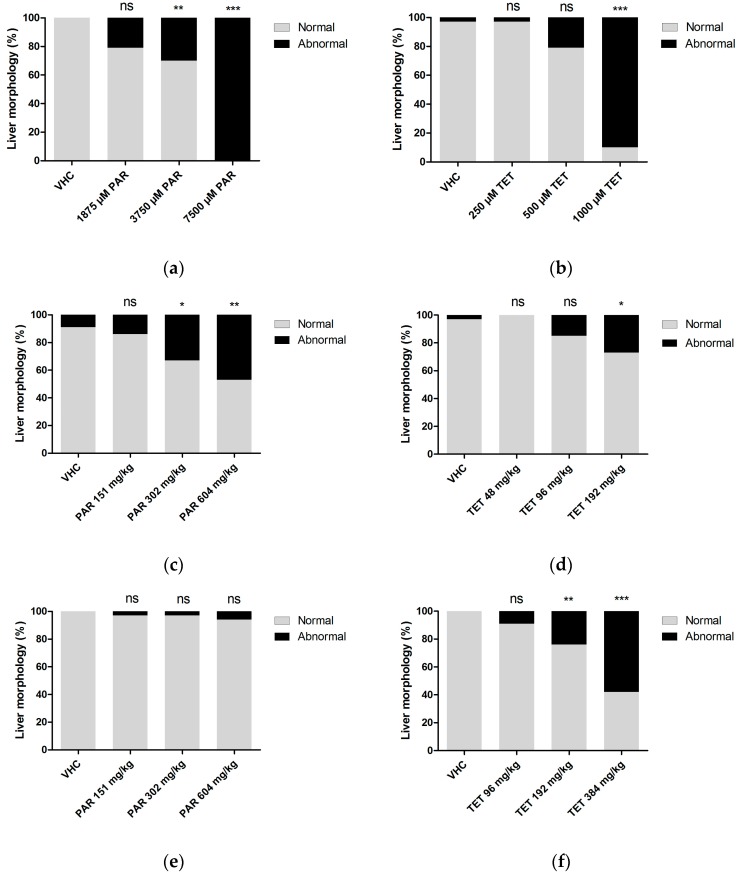
Hepatotoxicity assessed by visual analysis of liver morphology (by researchers blinded for the treatment conditions) using fluorescence microscopy after immersion (**a**,**b**); yolk injection (**c**,**d**) and pericardial injection (**e**,**f**) of paracetamol (**a**,**c**,**e**) and tetracycline (**b**,**d**,**f**). Analysis was performed after three days of treatment (immersion) or three days after treatment (injection), at 6 dpf. A total of 10 and 12 larvae were used individually per condition for immersion and injections, respectively. The experiment was performed in triplicate and data were pooled. The results are expressed as the percentage of larvae having normal or abnormal liver morphology. The data were analyzed by Fisher’s exact test. ns: no statistically significant difference; *: *p* < 0.05; **: *p* < 0.01: ***: *p* < 0.001; VHC: vehicle control; PAR: paracetamol; TET: tetracycline.

First, we immersed or injected Tg(*fabp10a:DsRed*) zebrafish larvae with the hepatotoxicants at their MC/MD (and dilutions) at 3 dpf and assessed their hepatotoxic effects after three days of treatment (immersion) or three days after treatment (injection) by visual analysis of liver morphology using fluorescence microscopy. We observed a range of abnormal liver phenotypes including reduced and enlarged livers (Figure 2). Tetracycline induced abnormal liver morphology in a dose-dependent manner in all three administration routes. Similar results were obtained with paracetamol, although no significant toxic effects were observed after pericardial injections (Figure 3). The non-hepatotoxic compounds (sucrose, rutin) did not induce abnormal liver morphology by any of the administration routes (results not shown). 

### 2.3. Hepatotoxicity Assessed by Size Analysis of Fluorescent 2D Liver Images 

**Figure 4:**
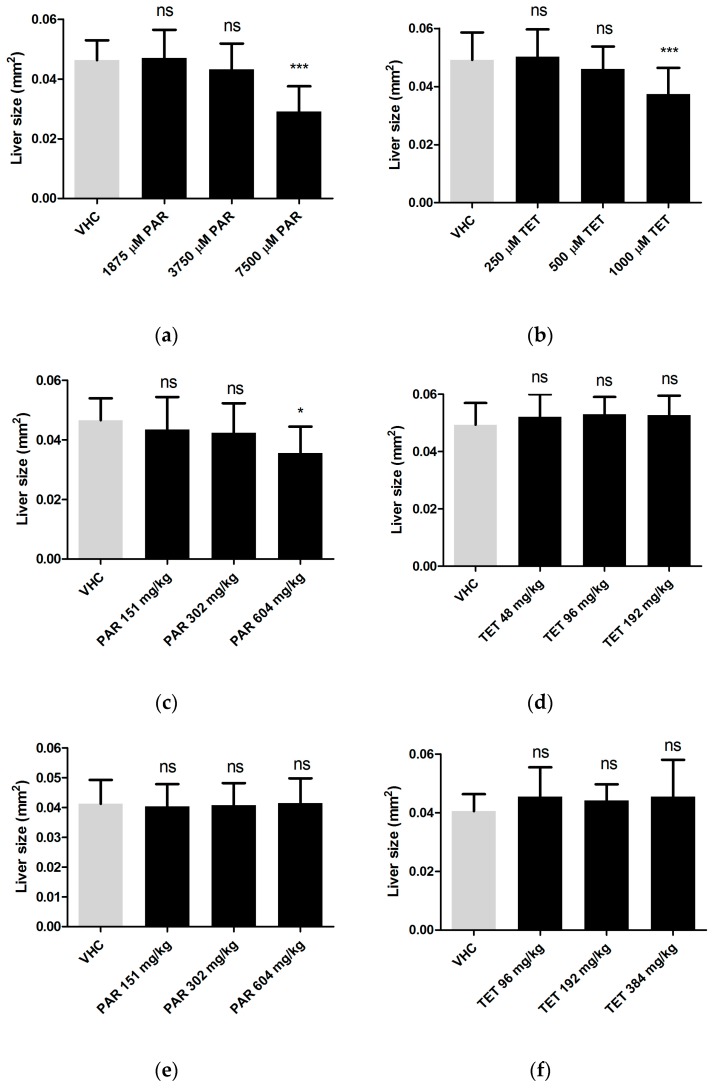
Hepatotoxicity assessed by size analysis of fluorescent 2D liver images after immersion (**a**,**b**); yolk injection (**c**,**d**) and pericardial injection (**e**,**f**) of paracetamol (**a**,**c**,**e**) and tetracycline (**b**,**d**,**f**). Analysis was performed after three days of treatment (immersion) or three days after treatment (injection), at 6 dpf. A total of 10 and 12 larvae were used individually per condition for immersion and injections, respectively. The experiment was performed in triplicate and data were pooled. The data were analyzed using one-way ANOVA. ns: no statistically significant difference; *: *p* < 0.05, ***: *p* < 0.001; VHC: vehicle control; PAR: paracetamol; TET: tetracycline. Means ± SD are shown.

Next, the 2D images obtained in the previous section were used to analyze the size of the fluorescent livers by ImageJ software. The results show that paracetamol and tetracycline were hepatotoxic only at their MC and not at lower concentrations. In case of injected hepatotoxicants, only paracetamol exhibited an effect at its MD after yolk administration (Figure 4). The non-hepatotoxic compounds (sucrose, rutin) did not induce any change in liver size as compared to livers of non-treated animals by any of the administration routes (results not shown).

### 2.4. Quantification of Recovered Hepatocytes by Cell Imaging Counting (CIC)

**Figure 5:**
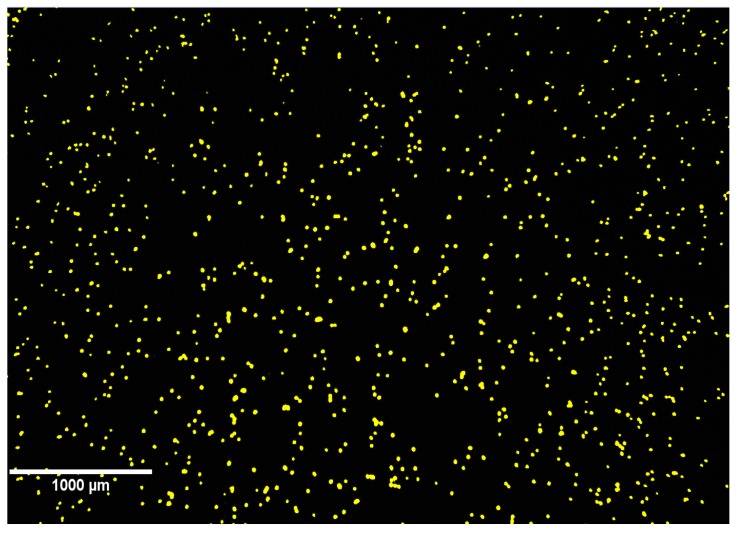
DsRed-labeled hepatocytes captured by cell imaging counting (CIC). Yellow dots represent single hepatocytes.

In order to use CIC for ex vivo quantification of the number of hepatocytes, we used a transgenic line Tg(*fabp10a:DsRed*) expressing DsRed in hepatocytes under the *fabp10a* promoter. To dissociate and recover hepatocytes from the larvae we investigated the effect of different common proteases (trypsin, collagenase and dispase) and mechanical forces (shaking, pipetting, and needle/syringe). We found that dispase, in combination with a gentle up and down pipetting of the digested larvae, resulted in a high recovery of well-separated and viable hepatocytes (Figure 5), whereas the other enzymes were not effective (collagenase) or highly toxic (trypsin) to the dissociated cells (results not shown).

To demonstrate the reproducibility of the method we quantified hepatocytes isolated from 6 dpf larvae (*n* = 80) incubated between 3 and 6 dpf with 1% dimethyl sulfoxide (DMSO) (vehicle control). The procedure was independently repeated three times, and the data were pooled. The average number of hepatocytes recovered amounted to 1509 ± 328 (average ± standard deviation (SD)) per larva (*n* = 240).

### 2.5. Hepatotoxicity Assessed by Quantification of DsRed-Labeled Hepatocytes Using Cell Imaging Counting (CIC)

**Figure 6:**
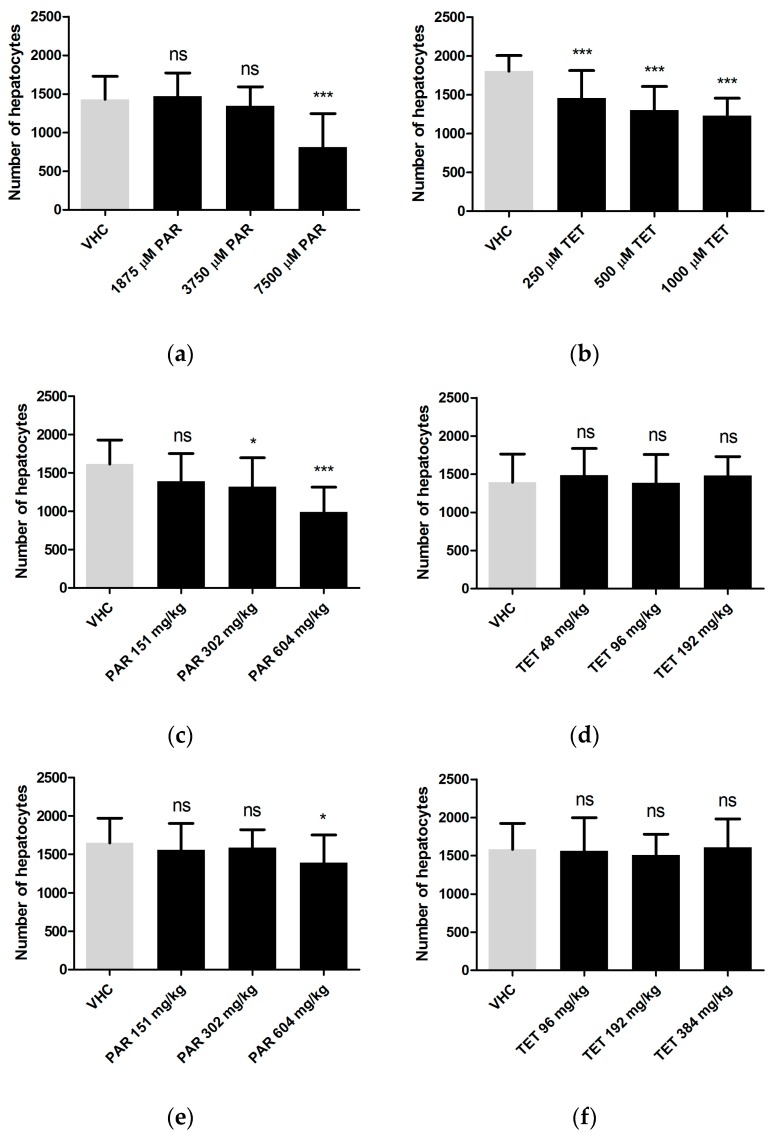
Hepatotoxicity assessed by quantification of DsRed-labeled hepatocytes using a CIC after immersion (**a**,**b**); yolk injection (**c**,**d**) and pericardial injection (**e**,**f**) of paracetamol (**a**,**c**,**e**) and tetracycline (**b**,**d**,**f**). Analysis was performed after three days of treatment (immersion) or three days after treatment (injection), at 6 dpf. A total of 10 and 12 larvae were used individually per condition for immersion and injections, respectively. The experiment was performed in triplicate and data were pooled. The data were analyzed using one-way ANOVA. ns: no statistically significant difference; *: *p* < 0.05; ***: *p* < 0.001; VHC: vehicle control; PAR: paracetamol; TET: tetracycline. Means ± SD of the number of hepatocytes per larva are shown.

We then used CIC and quantified ex vivo DsRed-labeled hepatocytes after dispase-digestion of hepatotoxicant-exposed larvae. The results show that this approach detected hepatotoxic effects induced by all three tetracycline concentrations. In case of paracetamol, only the MC triggered hepatotoxicity. As far as the injected doses are concerned, tetracycline showed no effects, whereas paracetamol was hepatotoxic in case of yolk administration (two concentrations) but also in case of the pericardial injection (only at MD) (Figure 6).

The non-hepatotoxic compounds (sucrose, rutin) did not induce any change in the amount of recovered hepatocytes as compared to the amount coming from livers of non-treated animals by any of the administration routes (results not shown).

### 2.6. Pharmacokinetics of Hepatotoxicants in Zebrafish Larvae

**Figure 7:**
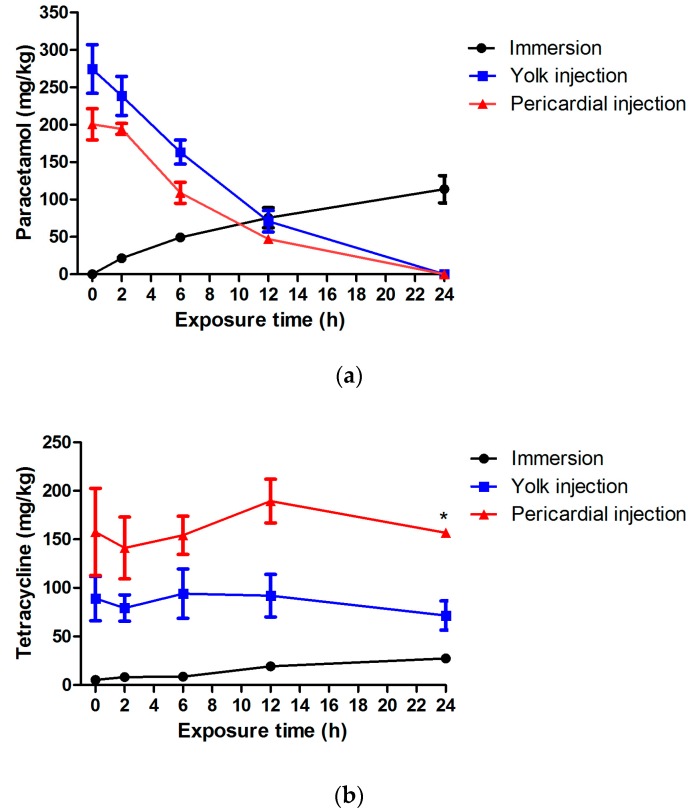
Pharmacokinetics after immersion, yolk and pericardial injection of paracetamol (**a**) and tetracycline (**b**). The compounds in larval extracts were quantified by ultra-high performance liquid chromatography with ultraviolet detection (UHPLC-UV) as a function of time (0–24 h post treatment). *n* = 3, *: consists of two measurements. Means ± SD are shown.

In order to investigate the pharmacokinetics of paracetamol and tetracycline after immersion or injection at their MC/MT, we quantified the compounds in larval extracts as a function of time (0–24 h post treatment) using ultra-high performance liquid chromatography with ultraviolet detection (UHPLC-UV). The validation data of the extraction and chromatography method can be found in the Appendix. The results show that in the case of immersion, the uptake of the compounds increased gradually as a function of time (Figure 7). Compared to tetracycline, paracetamol reached higher larval concentrations after 24 h, in line with the higher immersion concentrations used (1000 and 7500 µM, respectively). After both the yolk and pericardial injections (604 mg/kg), larval concentrations of paracetamol decreased over time with a half-life of 6–8 h. The compound was fully excreted or metabolized after 24 h. In contrast, larval tetracycline remained nearly constant in the 0–24 h period after injection. The amounts recovered after pericardial injections were about the double as compared to the quantity found after yolk injections. This is in agreement with the amount of tetracycline injected, i.e., 384 and 192 mg/kg, respectively).

## 3. Discussion

The first objective of this study was to evaluate cell imaging counting (CIC) as a new and efficient approach for ex vivo hepatotoxicity assessment in zebrafish larvae. CIC is a high-content imaging and quantification tool that is used for detecting cells and even changes in cells at a subcellular level [13,14]. Using a transgenic zebrafish line that specifically expresses fluorescent DsRed in the hepatocytes, we were able to quantify the amount of recovered hepatocytes in a reproducible way, even after a simple protease-digestion of the larvae in combination with a short centrifugation step. Indeed, in contrast to FACS analysis of cells recovered from whole organisms that needs sample filtering in order to prevent lines from clogging [11], our CIC-based method allows the straightforward detection of a limited number of fluorescent cells in the midst of non-fluorescent debris, without the need for time-consuming purification steps. This implies that the method can be used to test larger collections of compounds in a medium-throughput way. 

To evaluate the sensitivity of CIC to detect drug-induced hepatotoxicity, visual analysis of liver morphology by fluorescence microscopy and size analysis of fluorescent 2D liver images were employed as reference methods. The data show that all three methods were able to identify the hepatotoxic effects of paracetamol and tetracycline, at least when larvae were immersed in the non-lethal maximal concentration (MC) (Table 4). As compared to the other methods, CIC was more sensitive in detecting the effects induced by tetracycline at concentrations lower than MC. CIC also identified hepatotoxicity induced by paracetamol at MD via both yolk injection and pericardial injection, whereas the other methods were only successful for yolk injections. However, liver morphology assessment turned out to be more sensitive than CIC, e.g., in case of pericardial or yolk injections of tetracycline (Table 4). In general, this study also suggests that by combining visual assessment of liver morphology and CIC, a proper strategy is obtained for a very successful screening of the hepatotoxicity of compounds. As a matter of fact, by combining these methods, both paracetamol and tetracycline are recognized as hepatotoxic compounds via any of the three administration routes. 

The reason underlying the differences in sensitivity of the three methodological approaches is presently not clear. Obviously, further studies including detailed microscopic analyses of liver sections are needed that would allow us to correlate the outcome, as observed in this study, with histological changes and cellular viability.

Herein we also explored the applicability of using injections for the delivery of compounds investigated for their hepatotoxic effects. In case of paracetamol a clear effect was detected after yolk injection at MD, as identified by all three methods. Thus hepatotoxicity can also be induced by a single overdose of paracetamol in zebrafish larvae, as observed for humans [15]. Tetracycline induced pronounced effects, both after yolk and pericardial injections, although the hepatotoxicity was detected only by visual analysis of liver morphology, and not by the other methods (Table 4). 

Overall, the outcome therefore shows that also injections, especially in case of administering compounds into the yolk, can produce significant results in the framework of hepatotoxicity testing. However, as the pharmacokinetic data indicate that both paracetamol and tetracycline are absorbed by immersion, leading to obvious liver effects, it is not clear from the present data that yolk injections could be advantageous over simple bathing of larvae in compound solutions. Obviously, the yolk administration route that can also be performed automatically [16] is particularly of interest when testing hepatotoxicity of compounds that are poorly absorbed, i.e., in case of highly hydrophilic or large compounds [12].

The pharmacokinetic profile of paracetamol shows that almost no compound could be recovered from the larvae 24 h after yolk injection, although the hepatotoxic effects detected at 6 dpf are similar to the immersion results. This seems to indicate that the injurious effects take place particularly during the first 24 h after administration (3–4 dpf). Alternatively, it is possible that metabolites like *N*-acetyl-p-benzoquinone imine (NAPQI) are formed (not tracked down in this study) that exert their effect over time. Of interest, NAPQI-GSH adduct (GSH is the reduced form of glutathion) has been found when paracetamol was incubated with liver microsomes of adult zebrafish, possibly as the result of the expression of cytochromes P450 (CYPs), CYP3A65 with contributions of CYP3C1 [17]. Of interest, the genes for these CYPs are expressed in embryonal zebrafish as well [18], although it is not known whether the protein concentrations reach functional levels.

Interestingly, the pharmacokinetic profiles after yolk or pericardial injection of paracetamol are somewhat similar, although the hepatotoxic effects induced were different. Likely, both administration routes result in a dissimilar overall distribution of the compound in organs and tissue, affecting the final hepatotoxic effect.

In case of injected tetracycline, different pharmacokinetic profiles were observed as compared to the ones seen after injection of paracetamol. The concentrations remained somewhat constant over the first 24 h after administration, and it looks like the compound is not readily excreted from the larval body. It is possible that tetracycline binds to a large extent and with high affinity to local tissue without being distributed over the rest of the larvae. Anyhow, the final hepatotoxicity induced by the different administration routes as detected by liver morphology analysis is surprisingly quite similar. In contrast, when using CIC to assess hepatotoxicity, immersion led to pronounced effects (all concentrations active), whereas no impact was observed after injection. Again, the overall outcome regarding tetracycline is likely the consequence of a complex interplay between distribution, metabolism and detection of hepatotoxicity. These first intriguing results regarding pharmacokinetics and organ toxicity in zebrafish larvae definitely demand further investigation, and it looks like detailed metabolism data are required to fully understand the results. However, this experimental work was beyond the scope of the present study.

## 4. Materials and Methods 

### 4.1. Chemicals

Paracetamol, tetracycline, sucrose and rutin were purchased from Sigma-Aldrich (St. Louis, MO, USA). 

Protease inhibitor cocktail (1.5× solution) was made by dissolving 1 tablet (cOmplete mini Protease Inhibitor Cocktail Tablets, Roche, Basel, Switzerland) in 6.5 mL Dulbecco’s phosphate-buffered saline (DPBS) (Life Technologies, Ledeberg, Belgium) without calcium and magnesium.

Dispase II (Sigma-Aldrich) was dissolved in DPBS (Life Technologies, Ledeberg, Belgium) without calcium and magnesium to make a 20 mg/mL stock solution.

UHPLC experiments were performed using ammonium formate purchased from Fluka (Buchs, Switzerland). Formic acid was obtained from Acros Organics (Geel, Belgium), acetonitrile and methanol was purchased from Fischer Scientific (Loughborough, UK). All reagents were of analytical grade or better. Ultrapure water was produced using a Millipore Milli-Q Gradient system (Milford, MA, USA). All pH modifications were done with a Metrohm 691 pH meter (Antwerp, Belgium).

### 4.2. Zebrafish

All zebrafish experiments carried out were approved by the Ethics Committee of the University of Leuven (approval number P154/2015, the start date: 1 October 2015, the end date: 30 September 2019) and by the Belgian Federal Department of Public Health, Food Safety & Environment (approval number LA1210261).

A transgenic zebrafish line expressing DsRed in hepatocytes under the *fabp10a* promoter (herein referred to as Tg(*fabp10a:DsRed*)) was created and raised in our laboratory [9]. Tg(*fabp10a:DsRed*) zebrafish (larvae and adults) were kept in standard conditions for zebrafish (28 °C, 14/10-h light/dark cycle). Tg(*fabp10a:DsRed*) adults from the third and fourth generation were mated (two males and three females per cage). Only embryos of good quality (fertilized, clear cytoplasm and symmetric cleavage) were selected for experiments and kept in petri dishes containing Danieau’s solution until compound exposure. All larvae were derived from the same spawns of eggs for statistical comparison between the control and treated groups.

### 4.3. Compound Administration

Compounds tested were dissolved in DMSO (stock solution) and then further diluted in Danieau’s solution with a final concentration of 1% DMSO or diluted with 0.9% NaCl solution (1:1 ratio) to obtain a working solution for immersion and yolk/pericardial injections, respectively. At 3 dpf, hatched Tg(*fabp10a:DsRed*) larvae were transferred to 24-well plates (Falcon^®^, Corning, Lasne, Belgium) with each well containing 10 larvae in 1 mL Danieau’s solution and the dissolved drug or the vehicle control for immersion. For yolk injections, and pericardial injections into the bloodstream via the common cardinal veins [19,20], 12 larvae were used for each condition, and each larva was injected with 1 nL of solution at 3 dpf.

### 4.4. Hepatotoxicity Assessed by Visual Analysis of Liver Morphology Using Fluorescence Microscopy and Size Analysis of Fluorescent 2D Liver Images 

At 6 dpf treated larvae were anesthetized by 0.5 mM tricaine in a 6 well-plate (Falcon^®^) and arranged so that they were lying on their right sides with their heads facing left. This enabled us to view the left lateral side of the larvae and the larger lobe of the liver that was scored in a blinded manner under a fluorescence microscope (Leica MZ10F-Dsred channel, 40× magnification, Leica Microsystems, Wetzlar, Germany). Livers were considered abnormal if their typical globular structure underwent gross morphological changes. Images of the livers (including a scale bar) were imported to ImageJ software (National Institutes of Health, Bethesda, MD, USA) for determining the liver size. To that end, the picture was set to scale (functions: analyze, set scale), the liver area extracted (functions: split channel, threshold) and the area calculated (function: analyze particles, settings: size = 0−infinity, circularity = 0.00−1.00). 

### 4.5. Quantification of Hepatocytes Using Cell Imaging Counting (CIC) 

#### 4.5.1. Larvae Digestion and Recovery of Hepatocytes

In order to digest the larvae and separate hepatocytes from the liver, larvae were transferred to 96 well-plates (Falcon^®^, Corning, Lasne, Belgium) with one larva per well and incubated with 200 µL 2.5 mg dispase/mL DPBS for 4 h. The proteolytic activity was stopped by adding 100 µL of 1.5× protease inhibitors cocktail solution. The hepatocytes were dispersed by pipetting up and down 20 times with a 1 mL automatic pipette. After digestion, hepatocytes were positioned at the bottom of the well by centrifugation at 200× *g* (Jouan CR412 centrifuge, Jouan, Saint-Herblain, France) for 15 min.

#### 4.5.2. Cell Imaging Counting (CIC) of Hepatocytes

Fluorescent hepatocytes present in the wells were imaged and counted on an IN Cell Analyzer 2000 using software IN Cell Investigator Developer Toolbox (GE Healthcare, Machelen, Belgium), with a 2×/0.1 NA lens, DsRed fluorescence channel, a 0.1-s exposure time, and the intensity segmentation (172-4095) set to ensure hepatocytes were recognized selectively. 

### 4.6. Pharmacokinetics Study of Hepatotoxicants

#### 4.6.1. Sample Preparation

At given time points (0, 2, 6, 12 and 24 h), treated larvae were collected and transferred to 1.5 mL Eppendorf^®^ tubes pre-filled with acid washed glass beads (diameter: 710–1180 µm, Sigma-Aldrich) and 270 µL of extraction medium (1:2 water:methanol, each containing 0.1% formic acid) was added (10 larvae per Eppendorf^®^ tube). Samples were centrifuged for 15 s at 14,100× *g* (Eppendorf Mini Spin Plus, Hamburg, Germany) to insure high homogenization efficiency. Subsequently, the samples were homogenized with ultrasonication (Diagenode Bioruptor Plus, Seraing, Belgium) at +4 °C and high energy input setting to ensure the highest possible disruption of the cells. The overall treatment time was 15 min spread over 30 cycles of 30 s treatment followed by pauses of 30 s in-between. Six samples were homogenized during each treatment cycle. Subsequently, treated samples were centrifuged at 14,100× *g* for 15 min and 200 µL of supernatant was transferred to an empty Eppendorf tube (1.5 mL) and stored on ice until all samples were processed. All samples were then placed into a vacuum oven (Model 1410, Sheldon Manufacturing Inc., Cornelius, OR, USA) and evaporated until dry at +45 °C during 45 min. The samples were then reconstituted with a mobile phase-like solution (6% acetonitrile in water), vortexed for 5 s, resuspended at least three times with a micropipette and sonicated (Branson 3510, Danbury, CT, USA) for 15 min. The reconstitution volume was chosen such that the linearity range of the method was not exceeded and is given in Table 5. Finally, samples were transferred into amber glass vials (Supelco, Bellefonte, PA, USA) with 250 µL glass inserts (Supelco) for further analysis on UHPLC-UV.

#### 4.6.2. Instrumental Analysis

An Agilent 1290 UHPLC system (Agilent, Waldbronn, Germany) consisting of an autosampler, quaternary pump, degasser and DAD-detector (diode array detector) was used for all uptake measurements. Data acquisition and peak processing was performed using OpenLAB CDS Chemstation Edition 01.04 software (Agilent). All calculations concerning the evaluation of recorded data were made in MS Excel (Microsoft Corporation, Seattle, WA, USA).

Compounds of interest were separated chromatographically on an Acquity BEH C18 column (100 mm × 2.1 mm, d_p_ (particle diameter) = 1.7 µm) from Waters Inc. (Milford, MA, USA) at a flow rate of 0.4 mL/min. Gradient elution was performed starting at 97:3 (*v*/*v*) 10 mM ammonium formate (pH 2.8 modified with formic acid):acetonitrile, and changed to 18:82 (*v*/*v*) 10 mM ammonium formate (pH 2.8):acetonitrile in 10.5 min. The injection volume (*V*_inj_) of each sample was 2 µL. After elution of the compounds, a cleaning step at 100% acetonitrile was applied for 1.3 min after which the column was re-equilibrated for 7.0 min at initial conditions. Paracetamol was detected at a wavelength of 254 nm, while a wavelength of 274 nm was used for the measurement of tetracycline.

The larval amount of hepatotoxicant (mg/kg) was calculated assuming the weight of a 3 or 4 dpf Tg(*fabp10a:DsRed*) zebrafish larva to be 0.25 mg.

### 4.7. Statistical Analysis

Differences between drug-treated groups and control vehicle control (VHC) regarding numbers of hepatocytes and liver sizes were analyzed for statistical significance using an ANOVA test. Regarding the liver morphology, Fisher’s exact test was used for the analysis. *p* < 0.05 was considered statistically significant.

## 5. Conclusions

We successfully applied CIC *ex vivo* to detect hepatotoxicity induced by paracetamol and tetracycline in our transgenic zebrafish model. The outcome was in general comparable with the results obtained using two reference methods, i.e., visual analysis of liver morphology by fluorescence microscopy and size analysis of fluorescent 2D liver images, although clear differences exist depending on the administration routes investigated, i.e., immersion, yolk and pericardial injections. In general, immersion of larvae resulted in more consistent results among the different hepatotoxicity tests.

Unlike the other methods, our CIC-dependent approach measures directly and in an unbiased way the effect of compounds on the number of hepatocytes in the liver, is quick and reliable, and therefore provides a novel and straightforward tool for screening hepatotoxicants in early stages of drug development. As CIC allows for high-content imaging, the system is also amenable to more detailed analysis of recovered cells, e.g., multiparametric cytotoxicity testing. In addition, the method is also suitable for testing of other organ-related toxicities subject to the organs and tissue expressing fluorescent proteins in transgenic zebrafish lines. 

## Figures and Tables

**Table 1:** Compound concentrations used for hepatotoxicity testing (immersion).

Compound	Immersion Concentration (µM)		
	¼ MC	½ MC	MC
Paracetamol	1875	3750	7500
Tetracycline	250	500	1000
Sucrose	2000	4000	8000 ^1^
Rutin	50	100	200 ^1^

^1^ The highest soluble concentration. MC: maximum non-toxic drug concentration.

**Table 2:** Compound doses used for hepatotoxicity testing (yolk injection).

Compound	Injection Dose (mg/kg)		
	¼ MD	½ MD	MD
Paracetamol	151	302	604 ^1^
Tetracycline	48	96	192
Sucrose	137	274	548 ^1^
Rutin	38	75	150 ^1^

^1^ The highest dose that can be injected without precipitation. MD: maximum non-toxic drug dose.

**Table 3:** Compound doses used for hepatotoxicity testing (pericardial injection).

Compound	Injection Dose (mg/kg)		
	¼ MD	½ MD	MD
Paracetamol	151	302	604 ^1^
Tetracycline	96	192	384 ^1^
Sucrose	137	274	548 ^1^
Rutin	38	75	150 ^1^

^1^ The highest dose that can be injected without precipitation.

**Table 4:** Detection of compound-induced hepatotoxicity by the three methods.

Method	Treatment								
	Immersion			Yolk Injection			Pericardial Injection		
	¼ MC	½ MC	MC	¼ MD	½ MD	MD	¼ MD	½ MD	MD
Paracetamol									
Morphology ^1^	NS	**S**	**S**	NS	**S**	**S**	NS	NS	NS
Liver size ^2^	NS	NS	**S**	NS	NS	**S**	NS	NS	NS
^#^ Hepatocytes ^3^	NS	NS	**S**	NS	**S**	**S**	NS	NS	**S**
Tetracycline									
Morphology ^1^	NS	NS	**S**	NS	NS	**S**	NS	**S**	**S**
Liver size ^2^	NS	NS	**S**	NS	NS	NS	NS	NS	NS
^#^ Hepatocytes ^3^	**S**	**S**	**S**	NS	NS	NS	NS	NS	NS

^1^ Visual analysis of liver morphology by fluorescence microscopy; ^2^ Size analysis of fluorescent 2D liver images; ^3^ Analysis of amount of DsRed-labeled hepatocytes by CIC; ^#^ number of; Bold S: statistically significant difference; NS: no statistically significant difference.

**Table 5:** Reconstitution volumes for different administration routes.

Administration Route	Reconstitution Volume (µL)	
	Paracetamol	Tetracycline
Immersion	50	50
Yolk injection	100	50
Pericardial injection	100	50

## Abbreviations

CIC	Cell imaging counting
Dpf	Days post-fertilization
HLQ	Highest limit of quantification
Hpf	Hours post-fertilization
Lfbp	Liver fatty acid binding protein
LOQ	Lowest limit of quantification
MC	Maximum non-toxic drug concentration
MD	Maximum non-toxic drug dose
MQC	Medium quantified concentration
RSD	Relative standard deviation
UHPLC	Ultra-high performance liquid chromatography
UV	Ultraviolet
VHC	Vehicle control

## Appendix A. Method Validation of Compound Quantification

### Appendix A.1. Method Recovery

To determine method recovery, a first set of evaporated samples was prepared drug-free and then spiked with 10 µg/mL of each compound dissolved in DMSO (as depicted in Section 4.3) during the reconstitution step (*V*_reconstitution_ = 200 µL). Those samples showed the response *A_x_*_1_ (area of compound), which was unaffected by the sample preparation, though still reflecting possible influences of matrix on the chromatographic separation. A second set of samples contained extraction medium spiked with the same concentration as in *A_x_*_1_ and underwent the described preparation procedure with an equal reconstitution volume of 200 µL. In this way, the obtained response *A_x_*_2_ was connected to the compound loss during sample preparation. Method recovery was then calculated as follows:

(A1) $$\mathrm{Recovery}\;\left[\text{\%}\right]=100\text{\% }\times \;\frac{A_{x2}}{A_{x1}}$$

The obtained recovery values of 140.4% for paracetamol and 107.2% for tetracycline (Table A1) were considered to be satisfactory by taking into account low relative standard deviation (RSD) values of 1.2% and 0.1% accordingly and thus high repeatability of the data.

**Table A1:** Recoveries of paracetamol and tetracycline in zebrafish larvae 4 days post-fertilization (dpf).

Compound	Recovery (%)	RSD (%)
Paracetamol	140.4	1.2
Tetracycline	107.2	0.1

### Appendix A.2. Ultra-High Performance Liquid Chromatography-Ultraviolet ((UHPLC-UV) Method Validation

The developed instrumental method was validated with respect to linearity, limit of quantification, matrix effects and carry-over. Precision was tested at three concentration points: lowest limit of quantification (LOQ), medium quantified concentration (MQC) and highest limit of quantification (HLQ), while repeatability was determined for two concentration points: LOQ and HLQ. Each concentration was injected three times (precision) or six times (repeatability) to obtain RSD values, respectively. RSD limits were set to 20% near LOQ and 15% at other concentration points following the latest guidelines for method validation [21].

### Appendix A.3. Linearity, LOQ

The determination of linearity was performed in standard solutions of 6% acetonitrile in water containing compounds of interest at least 8 calibration points, while the obtained peak areas were plotted versus known concentrations. Paracetamol was tested at 0.02, 0.16, 0.33, 0.75, 1.5, 3, 6, 12, and 24 µg/mL and tetracycline at 0.25, 0.5, 1, 2, 4, 8, 16, and 32 µg/mL to define a curve range complying to linear model with R^2^ ≥ 0.995. The lower limit of quantification (LOQ) was defined as the concentration point where the signal of the analyte was 10 times higher than the noise.

Linearity curves were recorded for paracetamol and tetracycline in standard solutions. The R^2^ value was 0.9998 or better for paracetamol in the range 0.02–24 µg/mL and for tetracycline in the range 0.25–32 µg/mL (Table A2), while starting concentration was chosen corresponding to LOQ. These LOQ values were found to be similar to others determined in earlier studies involving UHPLC-UV equipment [22].

**Table A2:** Linearity of paracetamol and tetracycline; observed matrix effects. LOQ: lower limit of quantification.

Compound	Linearity Range (µg/mL)	LOQ (µg/mL)	*R*²	Matrix Effect (%)
Paracetamol	0.02–24	0.02	1	101
Tetracycline	0.25–32	0.3	0.9998	102

### Appendix A.4. Precision and Repeatability

Intermediate precision was determined for three different concentrations (0.025, 12, and 24 µg/mL for paracetamol and 0.25, 12.8, and 32 µg/mL for tetracycline) prepared and analyzed on three different days for both compounds. Simultaneously to those measurements a repeatability study was performed, requiring three additional injections (*n* = 6) at LOQ and HLQ.

Method precision was investigated by executing three injections at three different concentration levels (LOQ, MQC, HLQ) on three different days in standard solutions. Experiments revealed the highest RSD values at 8.1% (Table A3), thus in line with the limits (RSD < 15%) mentioned before and showing good precision of the obtained method for paracetamol and tetracycline.

Two samples in standard solutions of both compounds prepared at different concentrations (LOQ, HLQ) were analyzed six times each to determine method repeatability. The highest RSD value of 4.5% was obtained for LOQ of paracetamol displaying good overall reproducibility of the method.

**Table A3:** Determined repeatability and intermediate precision values for paracetamol and tetracycline. HLQ: highest limit of quantification; MQC: medium quantified concentration.

Compound	Repeatability		Precision		
	LOQ (%)	HLQ (%)	LOQ (%)	MQC (%)	HLQ (%)
Paracetamol	4.5	0.3	4.6	0.4	0.4
Tetracycline	3.3	0.5	3.4	8.1	7.7

### Appendix A.5. Matrix Effects and Carry-Over

Matrix effects were investigated by spiking evaporated blank samples of 4-dpf larvae (*n* = 10) with concentrations of 1.5, 6, and 24 µg/mL for paracetamol and concentrations of 2, 8, and 32 µg/mL for tetracycline and comparing the slope of the obtained linearity curve to those in standard solutions. The same matrix behavior was assumed for 3-dpf and 6-dpf larvae. Carry-over was examined by injecting drug-free standards or zebrafish solutions after each concentration level during the linearity measurements.

No significant deviations between measured concentrations in standard solutions and spiked zebrafish samples could be detected (Table A2), allowing for validation of the developed method in standard solutions. Following these results, the same matrix behavior was assumed for 3 and 6-dpf zebrafish larvae. Carry-over examination in the drug-free standards or zebrafish solutions revealed no carry-over records for either compound at any concentration levels.
